# Determinants of healthcare providers’ satisfaction with electronic medical records system in the Ashanti region of Ghana: A multicenter cross-sectional survey

**DOI:** 10.1371/journal.pone.0339107

**Published:** 2026-10-08

**Authors:** Bernard Gyamfi Anane, Victoria Bam, Gertrude Boafo, Emmanuel Osei-Peprah, Jacob Tetteh

**Affiliations:** 1 School of Nursing and Midwifery, College of Health Sciences, Kwame Nkrumah University of Science and Technology, Ghana; 2 Effiduase Government Hospital, Ghana Health Service, Ashanti Region, Ghana; Kwame Nkrumah University of Science and Technology Faculty of Pharmacy and Pharmaceutical Sciences, GHANA

## Abstract

**Background:**

Despite the scale-up of the Lightwave Health Information Management System (LHIMS) in Ghana, evidence on healthcare providers’ satisfaction remains limited. This study assessed healthcare providers’ satisfaction with LHIMS and its determinants in the Ashanti Region, Ghana.

**Methods:**

A multicentre, analytical cross-sectional study was conducted among 436 healthcare providers at three selected healthcare facilities in the Ashanti Region from August to October 2024. Data were collected using a self-administered questionnaire and analyzed using IBM SPSS Statistics version 25. Descriptive statistics were used to summarize the data, and logistic regression analysis was performed to identify factors associated with satisfaction. Results were reported using adjusted odds ratios (AORs) with corresponding 95% confidence intervals (CIs), with significance set at p < 0.05.

**Results:**

Of the 436 healthcare providers enrolled, data from 374 were included in the final analysis. The mean age of the participants was 33 years (*SD* = 5.7), and 66% were female. Overall, 57% (95% CI: 52–62%) of participants were satisfied with LHIMS. Satisfaction was independently associated with good perceived information quality (AOR = 3.50, 95% CI: 2.10–5.85), good system quality (AOR = 2.28, 95% CI: 1.38–3.78) and good system usability (AOR = 1.96, 95% CI: 1.20–3.19). In addition, adequate computer access (AOR = 2.06, 95% CI: 1.23–3.45) and high eHealth literacy (AOR = 1.74, 95% CI: 1.05–2.89) were associated with higher satisfaction.

**Conclusion:**

Healthcare providers’ satisfaction with LHIMS was moderate and associated with both system- and user-related factors. Strengthening information and system quality, usability, computer access, and eHealth literacy are essential to enhancing provider satisfaction and supporting sustainable EMR systems implementation in Ghana.

## Introduction

Electronic Medical Record (EMR) systems are computerised platforms designed to collect, store, and manage patient health information, including sociodemographic details, medical history, laboratory results and hospitalization history, under strict security measures [1,2]. Although the concept of digital health records emerged several decades ago, widespread adoption accelerated in the 1990s and has since become central to strengthening global health systems [3]. EMR systems are now recognized as essential tools for health sector reforms, with widespread adoption across healthcare systems globally [4]. At the policy level, the WHO Global Strategy on Digital Health 2020–2025 and the Sustainable Development Goals (SDG 3.8 on universal health coverage) strongly position the EMR system as critical for modernising health systems and improving population health [5,6].

EMR systems provide important clinical and organizational benefits including improved adherence to treatment guidelines, reduced medication errors, enhanced communication among multidisciplinary teams, and timely access to patient information [1,6,7]. They also streamline clinical workflows, reduce reliance on paper-based records, and support evidence-based decision-making [7]. At the organisational level, EMR systems improve operational efficiency, reduce costs, and contribute to the sustainability of healthcare institutions, thereby enhancing patient trust and service uptake [8,9]. Consequently, international actors such as the World Health Organisation and the World Bank have actively promoted EMR adoption, particularly in Sub-Saharan Africa (SSA), to improve health service delivery and strengthen health system performance [5,10].

Despite these benefits, the implementation and sustainability of EMR systems remain challenging in resource-limited settings. Financial constraints, unreliable electricity and internet connectivity, shortages of skilled personnel, weak policy frameworks, poor coordination, and limited technical support pose significant barriers [11–13]. Additionally, user-related barriers—such as limited digital literacy, poor system usability, insufficient training, and minimal involvement of healthcare providers in system design—also reduce provider confidence and hinder sustained use of EMR systems [7]. Since healthcare providers are the primary users of EMR systems, their satisfaction plays a critical role in determining successful adoption, continued utilization, and long-term sustainability. However, despite increasing implementation globally, evidence on healthcare providers’ satisfaction and the influencing factors remains limited in many LMICs, particularly in SSA [6,10].

In Ghana, health facilities historically relied on paper-based records until the introduction of electronic systems such as the Hospital Administration Management System (HAMS), JMeD, and Zen Medic to improve efficiency, accuracy, and data management [14,15]. However, these systems were privatized, expensive and fragmented [16]. To improve interoperability, transition from the paper-based records and standardize digital health implementation, the Ministry of Health (MoH) introduced the National eHealth Strategy and, later, the Digital Health Roadmap, which identified the Lightwave Health Information Management System (LHIMS) as the national electronic medical record platform [14,15]. Since its implementation, LHIMS has expanded substantially across public health facilities nationwide, including teaching and regional hospitals, district hospitals, polyclinics, and health centres [17,18]. The system has also captured the health records of nearly 21 million Ghanaians [18]. However, implementation challenges persist, including inadequate infrastructure, inconsistent training, poor technical support, and unreliable electricity and internet connectivity [19,20]. Concerns have also been raised about the insufficient involvement of healthcare providers in system development and deployment, leading to key clinical functions not being adequately captured and undermining the system’s acceptability and sustainability [21].

The implementation of EMR systems has received increasing scholarly attention in Ghana. However, several studies have primarily focused on themes such as knowledge, implementation process, post-implementation challenges, and utilisation rather than healthcare providers’ satisfaction [19,22–24]. Furthermore, available satisfaction studies have focused mainly on patients [25] or health managers [21] rather than frontline healthcare providers. Existing provider-focused evidence is limited to the Central Region [26]. No known studies have examined healthcare providers’ satisfaction with EMR system in the Ashanti region, despite the region having one of the largest concentrations of healthcare facilities and extensive LHIMS implementation coverage in Ghana. This absence of provider-focused research in the Ashanti Region highlights important evidence and contextual gaps, underscoring the need for further research.

Given that healthcare providers’ satisfaction strongly influences the acceptance, utilization, and sustainability of EMR systems, understanding their satisfaction levels and the determinants is essential to strengthening digital health implementation. Therefore, this study assessed healthcare providers’ satisfaction with LHIMS and associated factors in the Ashanti Region of Ghana. The findings may inform strategies to improve implementation, enhance user satisfaction, and support the long-term sustainability of digital health systems in Ghana.

## Materials and methods

### Study design and period

A multicenter, analytical, cross-sectional study was conducted from 28^th^ August to 30^th^ October 2024. This design was appropriate, as it enabled the assessment of healthcare providers’ satisfaction with the LHIMS, their perceptions of its qualities, and the determinants of satisfaction at a single point in time. This design is consistent with approaches widely applied in health information research to establish statistical associations between system features and user outcomes, while minimising the influence of longitudinal variation [6,10,13]

### Study area

The study was conducted in public healthcare facilities implementing LHIMS in the Ashanti Region of Ghana. The Ashanti Region is one of Ghana’s sixteen administrative regions, with Kumasi as its capital. The region lies in the middle belt of the country, specifically between longitudes 0.15^°^W and 2.25°W and latitudes 5.50°N and 7.46°N. The region encompasses a total land surface area of 24,389 square kilometres, accounting for 10.2% of Ghana’s total land area [27]

The Ashanti region is one of Ghana’s most populous and socioeconomically vibrant regions. It serves as a central referral hub for the middle and northern belts of the country. Healthcare in the region is organised into a tiered system comprising a teaching hospital [1], a regional hospital [1], district hospitals [26], polyclinics [8], health centres (162), and community-based health planning and services (CHPS) compounds (181) and several private healthcare facilities. With over 1,800 health facilities, it is one of the most densely served areas in the country and plays a central role in national health service delivery [28]

As part of Ghana’s eHealth strategy, the Ashanti Region has played a central role in the progressive rollout of the Lightwave Health Information Management System (LHIMS), the national electronic medical records platform. Implementation followed a phased approach, with teaching and regional facilities being the first to adopt LHIMS (2019–2020), followed by district hospitals (2021–2022), and then primary-level facilities, such as polyclinics and health centres (2022–2023) [15]. This staged implementation created natural variation in LHIMS maturity across levels of care, making the region an ideal setting for assessing user satisfaction.

Three facilities representing different levels of healthcare delivery were selected for this study. Kumasi South Regional Hospital (KSRH), located in the Asokwa Municipality of Kumasi, serves as the designated regional hospital for the Ashanti Region. It provides specialised and referral services, supports medical and nursing training, and has a bed capacity of about 120, with departments covering internal medicine, surgery, paediatrics, obstetrics and gynaecology, and accident and emergency. It was one of the first regional hospitals to implement LHIMS, starting around 2019 [15]

Suntreso Government Hospital (SGH), situated in the Kumasi Metropolis, functions as a district hospital, providing outpatient and inpatient services that encompass general medicine, maternal and child health, surgery, and laboratory support. With a bed capacity of about 60, SGH plays a vital role in delivering secondary-level care to surrounding urban communities. LHIMS was introduced at SGH between 2021 and 2022, in line with the phased rollout to district-level facilities [15]

Asuofua Polyclinic, situated in the Atwima Nwabiagya District, is a primary-level facility that offers outpatient care, maternal and child health services, preventive care, and limited inpatient care. The polyclinic serves as the first point of contact for healthcare in its catchment area. LHIMS was implemented at Asuofua Polyclinic in 2022–2023, during the expansion to primary-level facilities, thereby linking grassroots care into Ghana’s national digital health infrastructure.

By selecting facilities from three levels of healthcare delivery, regional, district, and primary, the study ensured representativeness and captured diverse provider experiences with LHIMS across different institutional contexts. This design also enabled meaningful comparisons of satisfaction across facilities with varying mandates, capacities, and durations of exposure to the system.

### Study population, inclusion and exclusion criteria

The source population comprised all healthcare providers working in public health facilities in the Ashanti Region of Ghana. The study population consisted of healthcare providers employed in these facilities who actively used the LHIMS. Healthcare providers, defined as clinical staff directly engaged in patient care, are the frontline users of LHIMS for documentation, prescribing, dispensing, and managing laboratory results. Their satisfaction is central to the effective utilization and long-term sustainability.

Eligibility was restricted to providers who had password-protected access to LHIMS and were actively using it, ensuring direct experience with its functionality. Healthcare providers who were on leave (annual, maternity, or other types) during the study period, as confirmed on the human resources leave roster, were excluded. In addition, providers with less than three months of LHIMS experience were excluded to minimise bias arising from limited exposure and to ensure adequate familiarity with system functions.

### Sample size determination

The sample size was determined using Cochran’s formula for estimating a single population proportion [29] assuming a 95% confidence interval, 5% significance level (α) *(two-sided),* 5% margin of error, and an expected user satisfaction with EMR systems 84.8% [13]. To account for variability introduced by the multistage sampling design, a design effect (D_eff) of 2.0 was applied. A 10% adjustment was added to compensate for potential non-response. The final estimated sample size was 436.

The sample size (n) was calculated as follows;


$$\begin{array}{c} \text{n}\_\text{intial}=\;[\frac{Z_{\left(\text{1}-\frac{\alpha }{\text{2}}\right)}^{\text{2}}\;p\;q}{E^{\text{2}}}]\;\times \;D_{eff} \end{array}$$

$$\begin{array}{c} \text{n}\_\text{intial}=\;[\frac{{\text{1}.\text{9}\text{6}\;}^{\text{2}}\;\times \;\text{0}.\text{8}\text{4}\text{8}\times \;\text{0}.\text{1}\text{5}\text{2}}{{\text{0}.\text{0}\text{5}}^{\text{2}}}]\;\times \;\text{2}.\text{0}=\;\text{3}\text{9}\text{6} \end{array}$$

$$\begin{array}{c} 𝐧\_𝐟𝐢𝐧𝐚𝐥=\text{ 396}+\;(\text{0}.\text{10}\times \text{396})\;=\text{ 436} \end{array}$$

where **n_initial** is the estimated sample size, p is the proportion of user satisfaction, Z _**(1-**_ α/2) is the Z-statistic value at the 95% confidence level and 5% significance level (α = 0.05) for 2 tails, which is 1.96, E is the margin of error 5% (0.05), and $D_{eff}$ is the design effect of 2.0, and **n_final** is the final sample size.

### Sampling technique

A multistage stratified sampling technique was employed to ensure fair representation of healthcare providers using LHIMS across different levels of care. The process involved two stages: facility selection and participant selection.

### Selection of facilities

All healthcare facilities in the Ashanti Region with functional LHIMS were stratified into three levels of care: [1] tertiary and regional hospitals [2], district hospitals, and primary-level facilities. Tertiary and regional hospitals were grouped within the same sampling stratum because LHIMS implementation commenced concurrently at both levels in the region.

From each stratum, one facility was randomly selected using the RANDBETWEEN function of Microsoft Excel version 2020 to reflect both the level of care and the stage of LHIMS adoption. Accordingly, Kumasi South Regional Hospital (regional), Suntreso Government Hospital (district), and Asuofua Polyclinic (primary) were chosen for the study.

### Selection of participants

The Health Information Departments of the selected facilities provided rosters of healthcare providers with access to LHIMS. The rosters initially included 672 providers at Kumasi South Regional Hospital, 523 at Suntreso Government Hospital, and 232 at Asuofua Polyclinic. After applying the study’s eligibility criteria, the sampling frame was reduced to 645, 499, and 219 providers, respectively.

The total sample size of 436 participants was proportionally allocated across the three facilities based on the size of their eligible populations: 206 from Kumasi South Regional Hospital, 160 from Suntreso Government Hospital, and 70 from Asuofua Polyclinic.

Within each facility, simple random sampling was then applied. Unique identification numbers were assigned to all eligible providers, and random numbers were generated using the RAND function in Microsoft Excel to select participants. A reserve list (10–15% of the required sample per facility) was also prepared to replace non-responders after two unsuccessful contact attempts.

Finally, selected providers were approached in their offices or wards, and eligibility was reconfirmed; the objectives of the study were then explained. Written informed consent was obtained before questionnaire administration. A flow diagram of the sampling process is presented in Fig 1.

**Fig 1:**
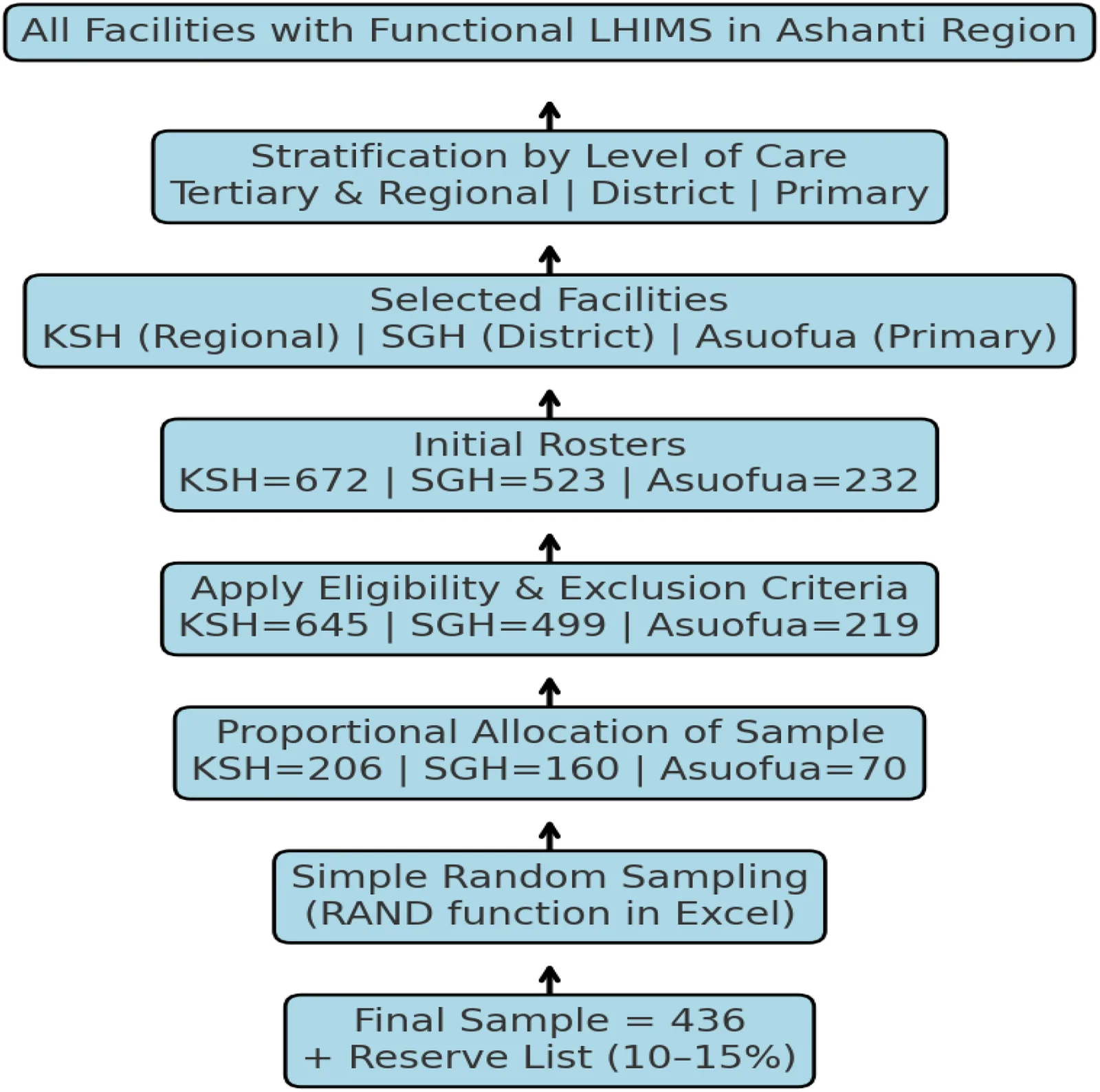
Flow diagram of the sampling process.

### Study variables

#### Dependent variable.

Healthcare providers’ satisfaction with LHIMS

#### Independent variables.

The independent variables comprised sociodemographic characteristics (age, gender, and years of professional experience, etc.), user-related factors (professional training, access to computers, and eHealth literacy), and system-related factors (system usability, system quality, service quality, and information quality).

### Instrumentation and measurement

A structured, self-administered questionnaire was used to collect data from healthcare providers. The instrument was developed using standardised, previously validated tools, including the eHealth Literacy Scale (eHEALS) [30] and the System Usability Scale (SUS) [31]. These were supplemented by items adapted from previous EMR studies [6,10,32] and by researcher-designed socio-demographic questions. The questionnaire was prepared in English and organised into the following sections: socio-demographic characteristics, user background, user satisfaction, and perceived technological qualities.

**Satisfaction with LHIMS** was assessed using seven items adapted from Dubale et al. [6] based on the user satisfaction construct of the DeLone and McLean Information Systems Success Model (D&M IS Success Model). Each item was scored on a 5-point Likert scale from 1 (strongly disagree) to 5 (strongly agree), yielding composite scores ranging from 7 to 35, with higher values indicating greater satisfaction. Previous studies categorized user satisfaction based on the distribution of composite scores [6,10]. Accordingly, we assessed the normality of composite scores using the Shapiro-Wilk test, which indicated a non-normal distribution (p < 0.05). Consequently, the median split was used to dichotomize participants into satisfied and dissatisfied groups, with scores ≥ the median classified as satisfied. Dubale et al. reported a Cronbach’s alpha value of 0.78.

**Professional training** was measured using one dichotomous item (yes/no) assessing whether healthcare providers had received training on LHIMS from the Health Information Department.

**Access to computers** was evaluated using one item, categorized as adequate, moderate, or inadequate.

The **8-item eHEALS** was used to assess **eHealth literacy [30]**. Each item was rated on a 5-point Likert scale (1 = strongly disagree to 5 = strongly agree), generating composite scores ranging from 8 to 40, with higher scores indicating better eHealth literacy. Composite eHEALS score ≥ 26 was classified as high eHealth literacy [6,33,34].

The **10-item SUS,** with items rated on a 5-point Likert scale (1 = strongly disagree to 5 = strongly agree), was used to measure **system usability [31]**. Positive items (1, 3, 5, 7, 9) were scored by subtracting 1 from the response, while negative items (2, 4, 6, 8, 10) were scored by subtracting the response from 5. Composite scores were multiplied by 2.5 to generate a total SUS score ranging from 0 to 100, with scores ≥68 indicating good usability [35,36]. The original SUS demonstrated a reliability coefficient of 0.90 [31]

**System quality**, **service quality**, and **information quality** were assessed using 22 items adapted from prior studies based on D&M IS Success Model [6,10]. System quality was measured using 6 items, service quality using 9 items and information quality using 7 items. All items were rated on a 5-point Likert scale, ranging from strongly disagree (1) to strongly agree (5). Composite scores ranged from 6 to 30 for system quality, 9–45 for service quality, and 7–35 for information quality, with higher scores indicating better perceived quality.

In the absence of universally accepted cut-off values, previous studies categorized information quality, system quality and service quality based on the distribution of composite scores [6,10]. Hence, the distribution of composite scores was measured using the Shapiro-Wilk test and demonstrated non-normality (p < 0.05). The median values were therefore used as the cut-off points to categorize participants into “good” and “poor” groups, with scores ≥ the median classified as “good perceived” quality

### Data collection procedures

Data were collected by six trained research assistants with backgrounds in nursing and physician assistantship, under the supervision of a medical officer, an MPhil nursing student, and the principal investigator *(two assistants and one supervisor at each facility).*

Eligible healthcare providers identified through the sampling procedure were contacted individually in their respective offices or wards. At initial contact, the purpose of the study, its objectives, and procedures were explained, and written informed consent was obtained prior to enrollment. To maintain anonymity during fieldwork, each participant was assigned a unique study identification number, which replaced names on all instruments.

Recruitment outcomes were documented in a logbook. Providers who declined to participate were recorded, and those who could not be reached after two separate attempts were classified as non-responders. Replacements were drawn sequentially from a pre-generated reserve list within the same facility. To minimise disruption to clinical duties, data collection was scheduled during less busy hours, and participants completed the questionnaire at a convenient time within their work units. Questionnaires were self-administered, with research assistants available to provide clarification when needed. Completed forms were checked on-site for accuracy and completeness, with immediate follow-up when omissions were found.

Supervisors provided on-site oversight across facilities and reviewed completed questionnaires on a daily basis. The research team held daily debriefing sessions to resolve challenges and harmonise procedures. At the end of each week, questionnaires were securely transferred to the principal investigator for storage and preparation for data entry.

### Quality assurance: Validity and reliability

The quality of the study instrument was assured through validity and reliability checks. Content validity was achieved by adopting standardised and widely used measures, including the eHealth Literacy Scale (eHEALS) and the System Usability Scale (SUS), supplemented with validated items adapted from the literature [6,10,30,31].

Face validity was confirmed by the Trainer of Trainees of the Lightwave e-Health Solutions, who reviewed the questionnaire for clarity, comprehensiveness, and contextual relevance.

A pretest was conducted at Effiduase Government Hospital to confirm applicability. Based on participant feedback, one item related to the District Health Information Management System (DHIMS) training was removed because it was limited to middle-level health managers and not associated with the utilization of LHIMS [37]

Construct validity was further examined using exploratory factor analysis (EFA). Prior to extraction, the Kaiser–Meyer–Olkin (KMO) measure of sampling adequacy and Bartlett’s test of sphericity were applied to confirm suitability for factor analysis. The KMO value was high (0.889), indicating that the data were suitable for factor analysis. Bartlett’s test of sphericity was statistically significant (p < 0.001), confirming that the correlation matrix was appropriate for factor extraction [38]. The EFA revealed that all constructs had eigenvalues greater than 1, and extracted factors explained a cumulative variance of 61.3%, exceeding the 50% minimum benchmark recommended in health sciences. Item loadings ranged from 0.39 to 0.64. The lowest loading (0.39) was slightly below the conventional cutoff of 0.40 but was retained due to its theoretical relevance and overall contribution to the construct. Most items exceeded the 0.40 threshold, indicating acceptable construct validity (See Table 1.)

**Table 1:** Validity and reliability of measurement tools.

statistic	Value
Kaiser-Meyer-Olkin (KMO)	0.889
Bartlett’s Test of Sphericity (p value)	<0.001
Number of Extracted components (Eigenvalue>1)	All constructs (multi-items)
Cumulative variance explained	61.3%
Range of Factor Loadings	0.385-0.640
Cronbach alpha	User satisfaction = 0.811, service quality = 0.869, information quality = 0.862, system quality = 0.747, system usability = 0.740. eHealth literacy = 0.651, computer literacy = 0.641

Reliability was assessed using Cronbach’s alpha to estimate internal consistency. Cronbach’s alpha values ranged from 0.64 to 0.87, indicating acceptable internal consistency for exploratory research [39](See Table 1.)

To enhance procedural reliability, all research assistants and supervisors underwent a two-day training session covering study objectives, ethical conduct, and standardised administration techniques before data collection commenced. Overall, the instruments were both valid and reliable

### Ethical considerations

Ethical approval for the study was granted by the Committee on Human Research, Publications, and Ethics (CHRPE) at the Kwame Nkrumah University of Science and Technology **(reference number: [CHRPE/AP/977/24])**. Administrative approval was obtained from the management of the selected hospitals before data collection.

Participation was entirely voluntary. Written informed consent was obtained from all participants after they were informed of the study objectives, procedures, potential risks, and benefits. Participants were informed of their right to decline or withdraw at any point without consequences.

To ensure confidentiality and anonymity, no personal identifiers, such as names, were recorded. Unique study identification numbers **(Questionnaire ID**) were used on all instruments. Completed questionnaires were stored securely in locked cabinets and later transferred to password-protected electronic databases accessible only to the research team.

The study posed minimal risk, as it involved no invasive procedures. The potential benefit lies in generating evidence that could inform improvements in the implementation of EMR systems and healthcare delivery.

### Data analysis

Data were entered and cleaned in Microsoft Excel 2020 for Mac and exported to IBM SPSS Statistics version 25 for analysis. Descriptive statistics were used to summarise sociodemographic characteristics, user-related factors, perceived system qualities, and satisfaction with LHIMS. Categorical variables were presented as frequencies and percentages, while continuous variables were summarised using means with standard deviations (SD) for normally distributed data, or medians with interquartile ranges (IQR) for skewed data.

Binary logistic regression analysis was conducted to identify the factors associated with satisfaction with LHIMS. A purposeful selection approach was adopted [40]. Bivariate logistic regression was initially performed to estimate crude odds ratios (COR) with 95% confidence intervals (CI). Variables with a p-value < 0.25 in the bivariate analysis were eligible for inclusion in the multivariable model, as this liberal cutoff helps reduce the likelihood of prematurely excluding potential predictors that may become significant after controlling for confounders [40]. Core theoretical constructs from the D&M IS Success Model were also selected regardless of their bivariate significance, as they represented key variables within the study framework.

Hierarchical logistic regression was used to assess the contribution of contextual variables beyond the theoretical constructs. Two models were specified. **Model 1** included the theory-driven constructs from the D&M IS success Model: system quality, information quality, service quality, training, computer literacy, and eHealth literacy, regardless of their bivariate significance. **Model 2**, the final explanatory model, extended Model 1 by incorporating contextual variables that showed at least a marginal association in the bivariate analysis (p < 0.25), including sociodemographic and professional characteristics (age, sex, cadre, and years of practice, etc.).

Model diagnostics included an assessment of multicollinearity using variance inflation factors (VIFs), with a cut-off of 5. “Facility level” and “computer access” were highly correlated, potentially introducing model instability and unreliable estimates [41]. Computer access was retained because it represented a more proximal and theoretically relevant construct of information system success [42]. Model fit was adequate, as indicated by the Hosmer–Lemeshow goodness-of-fit test (p = 0.537). The final model demonstrated good discriminative ability, with an area under the receiver operating characteristic curve (AUROC) of 0.784. These diagnostics confirmed that the final explanatory model was statistically robust and provided a reliable basis for identifying independent factors associated with user satisfaction.

Although a design effect was considered during sample size estimation, the final analysis was conducted at the individual provider level, with minimal clustering across facilities anticipated. Therefore, no adjustment for clustering was applied during the regression analysis.

Determinants were defined as variables that remained statistically significant in the final multivariate model at p < 0.05 and were presented as adjusted odds ratios (AORs) with corresponding 95% confidence intervals

## Results

### Study participants

A total of 1427 healthcare providers were assessed for eligibility, of whom 1,363 were eligible for participation. Among those excluded, 52 were on leave, and 12 had less than 3 months of LHIMS experience. Of the eligible healthcare providers, 436 were randomly sampled for inclusion in the study. However, data from 374 participants were included in the final analysis, yielding a response rate of 85.8%. Reasons for exclusion from analysis included incomplete questionnaires (n = 34), missing data on key variables (n = 24), and participant withdrawal (n = 4). Detailed results are presented in Fig 2.

**Fig 2:**
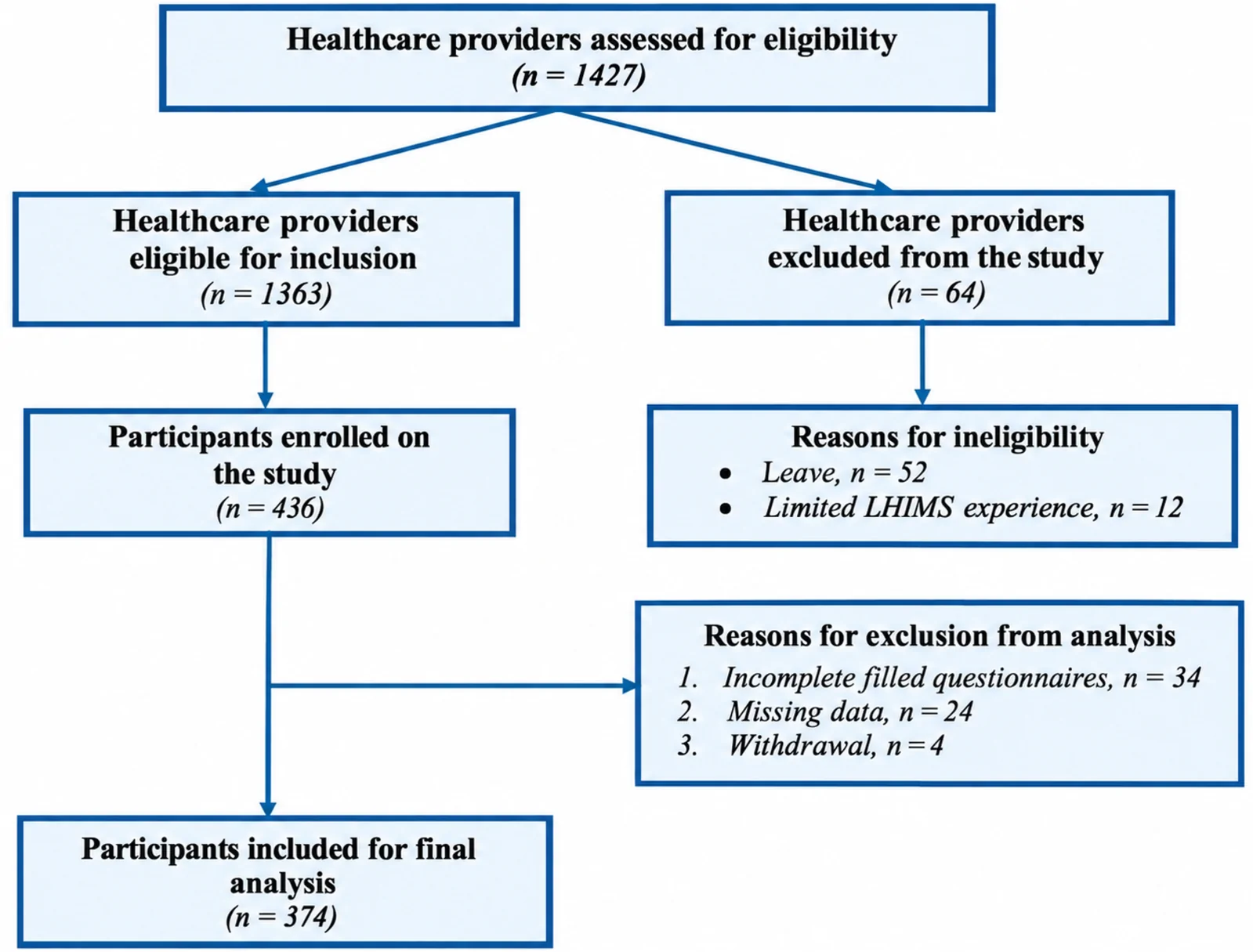
Study participants.

### Sociodemographic and professional characteristics of the healthcare providers (N=374)

Table 2 presents the socio-demographic and professional characteristics of the participants. The mean age was 33 years (*SD* = 5.70), two-thirds of participants (n = 247, 66%) were female, while more than half (n = 220, 58.8%) were married. Almost half of the participants (n = 183, 49%) were recruited from Kumasi South Regional Hospital, and nurses represented the largest professional group (n = 174, 46.5%).

**Table 2:** Sociodemographic and professional characteristics of the healthcare providers (N = 374).

Variable	Category	Frequency (n)	Percentage (%)
**Age (years)**			
*Mean age = 33 years (SD = 5.70)*	20-29 30-35 Above 35 years	111 119 144	29.6 32.0 38.4
**Gender**			
	Male Female	127 247	34.0 66.0
**Religion**			
	Christian Muslim	307 67	82.0 18.0
**Marital status**			
	Single Married Others (divorced, widowed)	145 220 9	38.8 58.8 2.40
**Professional category**			
	Nurse Midwives Doctor Pharmacist Other (PA, medilab, etc.)	174 94 54 35 17	46.5 25.1 14.4 9.5 4.5
**Years of Practice**			
*Mean = 6.33 (SD = 4.07)*	1-5 6-10 Above 10	195 120 59	52.1 32.1 15.8
**Health facility**			
	KSRH SGH Asuofua Polyclinic	183 128 63	49.0 34.0 17.0

*KSRH=Kumasi South Regional Hospital, SGH=Suntreso Government Hospital, PA=Physician Assistant, Medilab=Medical Laboratory Scientist*

### User background: Professional training, computer literacy, and proficiency

Table 3 presents results on user background. The majority of participants (n = 351, 94.0%) had received training on LHIMS, while exactly half (n = 188, 50%) reported having fair access to computers at their facility. Additionally, 262 (70%) demonstrated high computer literacy, whereas 199 (53.2%) had low eHealth literacy.

**Table 3:** User background: Professional training, computer literacy, and proficiency (N = 374).

Variable	Category	Frequency (n)	Percentage (%)
**Access to computers in your hospital**	Adequate Fair Inadequate	164 188 22	44.0 50.0 6.0
**LHIMS Training**	Yes No	351 23	94.0 6.0
**Computer literacy**	High Low	262 112	70.0 30.0
**eHealth Literacy**	High Low	175 199	46.8 53.2

High eHealth literacy= eHEALS ≥ 26.

### Healthcare providers’ satisfaction with LHIMS

The overall median satisfaction score was 29 (IQR: 27–32). Based on the median cut-off, 213 healthcare providers (57%, 95% CI: 52%–62%) were categorized as satisfied with the LHIMS.

Satisfaction varied across domains. The highest-rated domain was preference for the LHIMS over the paper-based records system (mean = 4.35, SD = 0.77). In contrast, the lowest rating was for the system’s impact on patient safety (mean = 4.06, SD = 0.72). Detailed results are presented in Table 4 and Fig 3.

**Table 4:** User satisfaction with lightwave health information management system (LHIMS) (N = 374).

Variable	Mean	SD
LHIMS help me to finish my work faster	4.27	0.61
My performance and productivity have improved due to LHIMS	4.07	0.71
The system has positive impact on quality of care	4.19	0.65
Patient’s safety has improved due to LHIMS	4.06	0.72
Information quality has improved due to LHIMS	4.22	0.72
LHIMS worth the time and effort required to use it	4.20	0.68
Prefer the LHIMS system to the paper record	4.35	0.77
Overall Users’ Satisfaction	**29.35**	**3.34**

**Fig 3:**
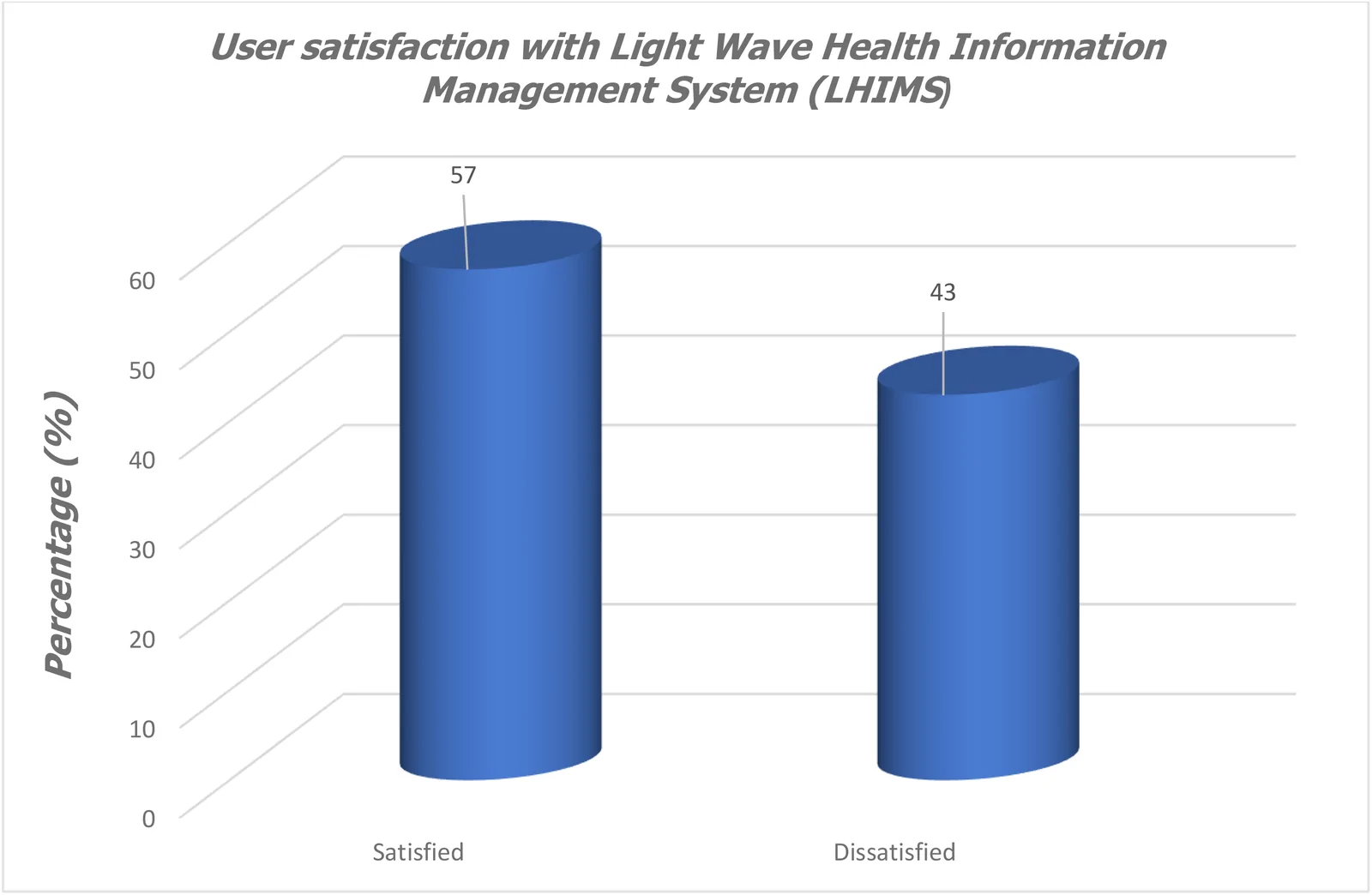
Overall Healthcare Providers’ Satisfaction with LHIMS (N = 374, Median = 29 (IQR = 27–32).

### Perceived qualities of the LHIMS

Table 5 presents results on healthcare providers’ perceived qualities of LHIMS. More than half of the participants rated system usability (n = 215, 57.5%), service quality (n = 196, 52.4%), and information quality (n = 194, 52%) as good, whilst approximately half (n = 188, 50.3%) rated system quality as good.

**Table 5:** Perceived qualities of the lightwave health information management system (LHIMS).

Variable	Median (IQR)	Category	Frequency (n)	Percentage (%)
System Usability	32 [(30–36)	Good	215	57.5
		Poor	159	42.5
System quality	25 [(23–27)]	Good	188	50.3
		Poor	186	49.7
Service Quality	34 [(30–37)]	Good	196	52.4
		Poor	178	47.6
Information Quality	29 (27–31)	Good	194	52.0
		Poor	180	48.0

Good perceived usability =SUS score ≥ 68, Good perceived quality=Composite scores ≥ the median

### Bivariate and multivariable analysis of the factors associated with healthcare providers’ satisfaction with LHIMS

In the bivariate analysis, access to computers, computer literacy, eHealth literacy, perceived information quality, perceived system quality, perceived service quality, and perceived system usability were all significantly associated with satisfaction at *p <* 0.05. Participants’ age range was significant at p < 0.25, thus included in the multivariable model

In the adjusted or multivariable model, perceived information quality, perceived system quality, perceived system usability, eHealth literacy, and computer access remained significantly associated with healthcare providers’ satisfaction with the LHIMS after adjusting for possible confounders. Good perceived information quality (AOR = 3.50, 95% CI: 2.10–5.85), good perceived system quality (AOR = 2.28, 95% CI: 1.38–3.78) and good perceived system usability (AOR = 1.96, 95% CI: 1.20–3.19) were independently associated with higher odds of healthcare providers’ satisfaction. Similarly, adequate access to computers (AOR = 2.06, 95% CI: 1.23–3.45) and high eHealth literacy (AOR = 1.74, 95% CI:1.05–2.89) independently increased the odds of healthcare providers’ satisfaction.

Although perceived service quality (COR = 1.98, 95% CI: 1.31–3.80) and computer literacy (COR = 2.52, 95% CI:1.60–4.00) were significant in the unadjusted analysis, these variables lost significance in the multivariable model (i.e., could not independently predict healthcare providers’ satisfaction after controlling for possible confounders). Detailed results are presented in (see Table 6).

**Table 6:** Bivariate and multivariable logistic regression analysis of the factors associated with user satisfaction with the LHIMS.

Variable	User satisfaction n (%)		COR (95% CI)	P-value	AOR (95% CI)	P-value
	Satisfied	Dissatisfied				
**Age Range**				**0.187**		
20-29 30-35 Above 35	69 60 83	42 59 60	0.62 (0.37-1.05) 0.51 (0.51-1.40) Reference (1)	0.074 0.507	0.87 (0.47-1.62) 0.58 (0.32-1.03)	0.658 0.064
**Gender**				**0.882**		
Male Female	73 140	54 107	1.03 (0.67-1.60) Reference (1)			
**Professional Category**				**0.336**		
Doctors Nurses Midwives Pharmacist Others	31 99 48 23 12	12 75 46 12 4	Reference (1) 1.02 (0.55-1.88) 0.81 (0.41-1.58) 1.48 (0.62-3.58) 2.32 (0.67-8.11)			
**Facility Level**				**<0.001**		
Regional District Primary	88 77 48	95 51 15	Reference (1) 2.63 (1.03-2.58) 3.46 (1.81-6.61)			
**Access to Computer**				**<0.001**		
Adequate Inadequate Fair	116 13 84	48 9 104	2.99 (1.92-4.66) 1.79 (0.73-4.39) Reference (1)	<0.001 0.204	2.06 (1.23-3.45) 1.85 (0.68-5.04) Reference (1)	0.006 0.231
**LHIMS Training**				**0.364**		
Yes No	202 11	149 12	1.50 (0.64-3.44) Reference (1)		1.18 (0.42-3.34) Reference (1)	0.865
**eHealth Literacy**				**<0.001**		
High Low	120 93	55 106	2.49 (1.63-3.80) Reference (1)		1.74 (1.05-2.89) Reference (1)	0.032
**Computer Literacy**				**<0.001**		
High Low	167 46	95 66	2.52 (1.60-4.00) Reference (1)		1.14 (0.66-1.97) Reference (1)	0.647
**System Quality**				**<0.001**		
Good Poor	138 75	50 111	4.09 (2.64-6.32) Reference (1)		2.28 (1.38-3.78) Reference (1)	0.001
**System Usability**				**0.001**		
Good Poor	138 75	77 84	2.00 (1.32-3.05) Reference (1)		1.96 (1.20-3.19) Reference (1)	0.007
**Service Quality**				**0.001**		
Good Poor	126 87	68 93	1.98 (1.31-3.80) Reference (1)		1.03 (0.61-1.75) Reference (1)	0.915
**Information Quality**				**0.001**		
Good Poor	148 65	48 113	5.36 (3.43-8.38) Reference (1)		3.50 (2.10-5.85) Reference (1)	0.001

Hosmer and Lemeshow = 0.537, AUROC = 0.784, 95% CI: 0.74–0.83, AOR = Adjusted Odds Ratio, COR = crude odds ratio

Others = medical laboratory scientists, herbal medicine, radiographers

## Discussion

### Healthcare providers’ satisfaction with LHIMS

This study found moderate satisfaction with LHIMS among healthcare providers. User-related factors, such as access to computers and eHealth literacy, and system-related factors, such as information quality, system quality, and system usability, were independently associated with satisfaction. The moderate satisfaction observed suggests that while LHIMS has gained some acceptance among healthcare providers, its full potential has not yet been realised. Similar moderate levels of satisfaction have been reported in Ghana and Ethiopia, where approximately half of healthcare providers expressed satisfaction with the implemented EMR system [6,26]. This similarity may reflect common infrastructural constraints and implementation realities across many SSA and LMICs, including inconsistent internet connectivity, frequent power interruptions, limited technical support, and inadequate computer access [21]. Methodological consistencies may also partly explain the comparable findings, as our study, Achampong et al. [26] in Ghana, and Dubale et al. [6]in Ethiopia, used similar validated instruments and scoring approaches to assess healthcare satisfaction with the EMR system.

Contrastingly, substantially higher satisfaction levels have been reported in higher-income countries (HICs) such as Saudi Arabia, the United States, and Canada, where healthcare institutions often benefit from stronger digital infrastructure, stable internet connectivity, reliable electricity supply, dedicated technical support teams, and sustained financial investment in health information technologies [43–45]. These enabling conditions make EMR use smoother, reduce operational frustrations, and improve user experiences, increasing satisfaction among healthcare providers.

Nevertheless, higher satisfaction levels have also been documented in some LMICs, including Rwanda and Malaysia [13,46]. The possible explanations include variations in system maturity, implementation duration, and institutional investment in digital health systems. In Rwanda and Malaysia, for instance, the implemented EMR systems had been operational for nearly a decade, allowing sufficient time for iterative system upgrades, repeated user training, and the adaptation of workflows to digital systems. In Malaysia, Azmi et al. [13] further emphasized that funding allocation for digital health had increased, leading to significant infrastructure strengthening and system upgrades, which likely explain the higher satisfaction rates. In contrast, LHIMS has been operational for less than 5 years in the selected facilities and may therefore still be in the mid-adoption phase, requiring further optimisation, sustained technical support, and continuous capacity building to achieve higher levels of satisfaction and long-term sustainability [15].

Variations were observed across specific satisfaction domains. Healthcare providers expressed greater satisfaction with the quality of information generated by LHIMS and showed a stronger preference for electronic records over paper-based documentation. Similar evidence has been reported in Norway, where EMR systems improved documentation quality and accessibility [47]. A possible explanation is that EMR system generate more structured, legible, and standardised clinical records than handwritten documentation, which is often difficult to interpret and prone to errors [48]. These features likely improve communication among healthcare providers and support more efficient clinical decision-making.

Satisfaction with efficiency was also high, as healthcare providers reported that LHIMS helped them complete their work faster. This finding is consistent with studies in Ethiopia and Saudi Arabia, where the use of EMR system improved efficiency by simplifying workflows and facilitating quicker access to patient information [5,10]. A plausible explanation is that once information is entered into the EMR system, it can be retrieved and reused instantly, unlike paper-based documentation, which requires repeated entries and manual searching of patients’ folders [47].

Conversely, lower satisfaction was observed in domains related to patient safety and quality of care. One important feature of LHIMS is its broad access to patient information across units, which facilitates continuity of care. Although this design improves accessibility and coordination, it may also raise concerns regarding confidentiality, data security, and patient trust. Similar governance and security concerns have been highlighted in Ghana and other African contexts, where weak data protection frameworks and limited accountability mechanisms undermine confidence in EMR systems [21,49]. Another possible explanation is that providers may spend prolonged periods navigating the system for documentation, retrieving laboratory results, and reviewing pharmacy instructions, thereby reducing the time available for direct patient care. This shift in focus from bedside interactions to computer-based tasks may limit provider-patient engagement and inadvertently contribute to delays or lapses in care delivery. Similar concerns have been documented in Ethiopia, where increased screen time and documentation demands negatively affected patient-provider communication and interactions [50,51]

Overall, these findings suggest that while LHIMS improves documentation quality and workflow efficiency, unresolved concerns about data security and reduced bedside engagement may limit its perceived contribution to patient safety and quality of care. These findings underscore the need for policymakers, hospital administrators, and system developers to strengthen the data governance framework, enforce role-based access controls and establish clear accountability mechanisms to protect patients’ confidentiality and trust in the system. Healthcare institutions should implement competence-based training and workflow redesign to reduce documentation burden, improve navigation, and promote provider-patient interactions during clinical encounters. Addressing these challenges may enhance healthcare providers’ confidence in the system and optimize the quality and safety benefits of LHIMS implementation in Ghana

### Determinants of healthcare providers’ satisfaction with LHIMS

#### User-related factors.

The study identified several user-related predictors of LHIMS. Adequate computer access was independently associated with higher satisfaction with LHIMS, suggesting that providers with easier access to computers are better positioned to integrate the EMR into routine clinical practice. Corroborative findings have been reported in Ethiopia and Malaysia, where adequate access to computers, such as availability of multiple workstations, ownership of personal laptops and other digital devices, improved healthcare providers’ satisfaction and utilization of implemented EMR systems [10,52]. This finding was expected because adequate computer access reduces workflow interruptions, device-sharing challenges, and delays in documentation and in retrieving patient information. These findings highlight the importance of equitably distributing digital resources across healthcare facilities to optimise use and enhance users’ satisfaction with LHIMS in Ghana.

eHealth literacy also emerged as an important associated factor of satisfaction. Providers with stronger digital competencies were more likely to report satisfaction, possibly because they could navigate the system more efficiently, interpret digital outputs accurately, and integrate electronic information into clinical decision-making. This finding is consistent with the eHealth Literacy Model and evidence from other studies, which have identified eHealth literacy as a driver of IS success [26,30,53,54]. However, contrasting findings have been reported. For instance, Dubale et al. [6] in Ethiopia observed that eHealth literacy lost its independent association with satisfaction in the multivariable model. This discrepancy may reflect differences in digital infrastructure, the scope of training interventions, and the methods used to measure eHealth literacy.

It is equally important to note that not all user-related variables were associated with satisfaction. For instance, LHIMS training did not emerge as a determinant of satisfaction. This finding is consistent with studies by Alasmary and Househ et al. [55] and Walle et al. [54], who similarly reported no significant relationship between basic technological training and satisfaction. In contrast, empirical studies, as well as established IT adoption frameworks such as the Technology Acceptance Model (TAM), have documented a positive, often strong association between training and satisfaction [6,10,13,56]. A plausible explanation for this apparent inconsistency lies in the training profile of the study population. Nearly all providers surveyed (94%) had already received training, leaving minimal variability for statistical discrimination. In such circumstances, where training coverage approaches universality, a ceiling effect occurs, thereby diminishing the explanatory power of training as a differentiating factor for satisfaction.

Similarly, computer literacy was not associated with user satisfaction, despite a substantial proportion of the providers demonstrating good literacy levels. Conversely, studies in Ethiopia, Saudi Arabia, and Australia reported a significant association between computer literacy and users’ satisfaction with the EMR system [6,32,54]. The possible explanation is that the measure of literacy used in this study may not have adequately captured the practical competencies required for efficient EMR system use, such as typing speed, multitasking ability, and navigating efficiency. Although many providers were familiar with the EMR system, operational challenges, including limited typing skills and inefficient navigation, remained widespread and may not have translated into measurable differences in satisfaction.

Overall, these findings suggest that satisfaction with LHIMS depends less on foundational computer knowledge and skills alone and more on the availability of enabling resources and advanced digital competencies. It therefore underscores the need for healthcare institutions and policymakers to move beyond one-time introductory programmes towards continuous, competency-based digital capacity-building initiatives. Such interventions should incorporate hands-on practical sessions, workflow-oriented coaching, and tailored support aimed at strengthening operational skills with advanced EMR systems. Additionally, improving access to functional computers and supportive digital infrastructure may further enhance user experience and long-term satisfaction with the EMR system.

### System-related factors

Several system-related constructs also predicted satisfaction. System quality was independently associated with user satisfaction, with providers who perceived LHIMS as stable, responsive, and user-friendly reporting higher satisfaction. This finding resonates with evidence from Tanzania and Greece [57,58]. A possible explanation is that reliable systems inspire user confidence, reduce workflow interruptions, and enable providers to focus on patient care rather than troubleshooting technical issues.

System usability was a predictor of providers’ satisfaction with LHIMS. Providers who perceived LHIMS as simple, consistent, and easy to navigate were better positioned to report positive experiences with the system. Similar findings have been reported in Ghana and Ethiopia [6,10]. Usable systems reduce the cognitive burden on providers, shorten the learning curve, and fit more smoothly into clinical routines. Conversely, challenges such as navigating multiple tabs to retrieve patient information may increase workload and frustration, thereby reducing satisfaction with the system.

Information quality similarly played a critical role in predicting satisfaction. Providers who perceived system outputs as precise, accurate, and valid reported higher satisfaction. Consistently, studies in SSA settings, including Ghana, Ethiopia, and Nigeria, as well as in Brazil, have reported a positive correlation between information quality and user satisfaction with EMR systems [6,26,59,60]. The D&M IS Success Model similarly positions information quality as an essential determinant of information system success [42]. EMR systems produce well-structured, legible documentation that is easier to read and interpret. This improves communication among providers, reduces duplication of work, and enhances clinical decision-making, thereby increasing user satisfaction.

Service quality did not significantly predict satisfaction in this study, despite being a central construct of the D&M IS Success Model. Similar findings have been reported in Ethiopia and Rwanda [46,54]. However, studies from higher-resource contexts such as Canada, Saudi Arabia, and Brazil have demonstrated that service quality characterised by responsive technical support influences EMR satisfaction [60–62]. A plausible explanation is that infrastructural constraints common in low-resource settings, such as unstable electricity, poor internet connectivity, and prolonged downtime, tend to overshadow perceptions of service quality, thereby weakening its predictive role [46]. In Ghana, these systemic challenges may be compounded by concerns from health leaders that providers were not adequately consulted during the design and implementation of LHIMS, and that their inputs were not duly incorporated [21]. These contextual issues may therefore reduce the explanatory power of service quality within the D&M IS Success Model, as structural barriers and limited participatory design weaken its potential impact on user satisfaction.

Overall, our findings suggest that system-related attributes, particularly quality, usability, and information outputs, are central to provider satisfaction with LHIMS. These findings underscore the importance of designing user-centred systems that align with the workflow realities and operational demands of clinical care. Optimizing system performance, improving usability, and ensuring high-quality, reliable information outputs are therefore critical to strengthening user confidence and satisfaction, and to promoting sustained system acceptance. Consequently, policymakers, health facility managers, and system developers should actively involve frontline healthcare providers in the design, upgrading, and implementation of EMR systems to ensure improvements are responsive to users’ practical needs and experiences.

### Limitations and strengths

This study has certain limitations. First, reliance on self-reported measures may have introduced response and social desirability bias, as healthcare providers may be inclined to give socially acceptable or professionally favourable responses about their experiences with LHIMS, potentially leading to an overestimation of satisfaction and positive perceptions of the system.

Furthermore, the cross-sectional design limits causal inference. This is because the identified determinants (exposures) and healthcare providers’ satisfaction with LHIMS (outcome) were assessed simultaneously, making it difficult to confirm temporality and directionality.

In addition, the study was conducted in public healthcare facilities in the Ashanti region, which may limit the generalizability of findings to private healthcare facilities or other regions with different infrastructural and implementation contexts. Future longitudinal and qualitative studies, incorporating objective system measures, such as system log files, error rates, or task completion times, may provide deeper insights into healthcare providers’ experiences and changes in satisfaction over time.

Despite these limitations, the study has notable strengths. The use of validated, theory-driven instruments grounded in the D&M IS Success Model enhanced the credibility and theoretical rigour of the findings. Moreover, the inclusion of facilities across primary, district, and regional levels, combined with a multistage sampling approach using stratified proportional allocation and an adjusted design effect, improved representativeness, minimised clustering bias, and ensured methodological rigour. These methodological strengths, combined with the diversity of study settings, provide reliable evidence to inform digital health policy and practice in Ghana

## Conclusion

This study found that healthcare providers’ satisfaction with LHIMS in Ghana was moderate. Satisfaction was associated with both user-and system-related factors, particularly computer access, eHealth literacy, system quality, usability, and information quality. These findings suggest that improving provider satisfaction with LHIMS requires not only strengthening technological infrastructure but also enhancing users’ digital capacity and optimizing system performance.

Policymakers and hospital administrators should prioritise reliable internet connectivity, access to computers, user-centred system design and continuous digital skills training for healthcare providers. System developers should collaborate and incorporate end-user feedback into future system upgrades to further improve the usability and long-term sustainability of LHIMS. These findings provide important evidence to inform strategies aimed at improving the implementation, usability and sustainability of EMR systems in Ghana and similar-resourced limited settings.

Future studies should consider longitudinal, implementation-focused, or mixed-methods designs to better explore causal relationships and gain deeper insights into healthcare providers’ experiences of EMR system implementation and use. Future research should also examine the impact of EMR systems on patient outcomes and quality of care in resource-limited settings. In addition, studies involving private healthcare facilities and other regions in Ghana would also help improve the generalisability of findings and provide broader evidence to guide EMR system implementations in similar resource-limited settings.

## Supporting information

S1 Text Data collection instrument. (DOCX)

S1 Dataset Raw data. (SAV)
