# Supplementary material for: Determinants of healthcare providers’ satisfaction with electronic medical records system in the Ashanti region of Ghana: A multicenter cross-sectional survey
Source: PLoS One. 2026 Oct 8;21(10):e0339107. doi: 10.1371/journal.pone.0339107 (PMC13649020; doi:10.1371/journal.pone.0339107)
Supplement: Supplementary file 1 [file pone.0339107.s001.docx]

**Data Collection Tool**

**QUESTIONNAIRE**

Dear Respondent,

This is a data collection tool (questionnaire) on a study titled “**Determinants of Healthcare Providers’ Satisfaction with Electronic Medical Records System**

**in the Ashanti Region of Ghana*”.***

Thank you for volunteering to complete this questionnaire. Your response is very important. The purpose of this questionnaire is purely academic; therefore, all your responses will be treated with strict confidentiality and will in no way be linked to you.

Thank You.

**INSTRUCTIONS:**

- Where alternatives have been provided, tick (✓) your answer in the spaces provided.
- For the other questions without alternatives at all, kindly write your answers in the spaces provided.
- For sections D, E, and F: Indicate your level of agreement with each statement using a five-point Likert Scale of Strongly disagree (SD), Disagree (D), neutral (N), Agree (A), and strongly agree (SA)

**SECTION A: SOCIO-DEMOGRAPHIC INFORMATION**

| Variable | Response |
| --- | --- |
| 1. How old are you? | ……………………………..(Years) |
| 2. Gender | ☐Male  ☐Female |
| 3. Religion | ☐Christian  ☐Muslim |
| 4. Marital status | ☐Single  ☐Married  ☐Divorced/separated  ☐If other specify……………………… |
| 5. Professional category | ☐Nurse  ☐Midwife  ☐Medical officer  ☐Pharmacist/Dispensary officer  ☐Laboratory scientist  ☐ If other specify……………… |
| 6. Working Experience (Years) | ……………………….. |
| 7. Health facility | ☐ Kumasi South Hospital  ☐ Suntreso Government Hospital  ☐ Asuofua Polyclinic |

**SECTION B: PROFESSIONAL TRAINING, INFORMATION TECHNOLOGY (IT) QUALIFICATION, AND EXPERIENCE**

| Variable | Response |
| --- | --- |
| 8. How do you rate access to computers in your hospital? | ☐Adequate  ☐Fair  ☐Inadequate |
| 9. Have you taken EMR training? | ☐Yes  ☐No |

**SECTION C: USER SATISFACTION**

| Statement | Level of agreement | | | | |
| --- | --- | --- | --- | --- | --- |
|  | Strongly agree | Agree | Neutral | Disagree | Strongly disagree |
| 10. The EMR system help me to finish my work faster |  |  |  |  |  |
| 11. My performance and productivity have improved due to the EMR system |  |  |  |  |  |
| 12. The system has positive impact on quality of care |  |  |  |  |  |
| 13. Patient’s safety has improved due to the EMR system |  |  |  |  |  |
| 14. Information quality has improved due to the EMR system |  |  |  |  |  |
| 15. The EMR system worth the time and effort required to use it |  |  |  |  |  |
| 16. I prefer the EMR system to the paper record |  |  |  |  |  |

**SECTION D: COMPUTER LITERACY**

| Statement | Level of agreement | | | | | |
| --- | --- | --- | --- | --- | --- | --- |
|  | Strongly agree | Agree | Neutral | Disagree | | Strongly disagree |
| 17. I am interested in working with computers |  |  |  |  |  | |
| 18. I have moderate skill in using computers |  |  |  |  |  | |
| 19. I take computer trainings in the hospital |  |  |  |  |  | |
| 20. I feel that using computers will support me to be more efficient in the future |  |  |  |  |  | |

**SECTION E: SYSTEM QUALITY**

| Statement | Level of agreement | | | | |
| --- | --- | --- | --- | --- | --- |
|  | Strongly agree | Agree | Neutral | Disagree | Strongly disagree |
| 21. The system is easy to learn |  |  |  |  |  |
| 22. The system is user friendly |  |  |  |  |  |
| 23. The system is stable |  |  |  |  |  |
| 24. I can retrieve information easily |  |  |  |  |  |
| 25. The system responds faster and promptly to queries |  |  |  |  |  |
| 26. Patient data transfer between department is easy |  |  |  |  |  |

**SECTION F: SYSTEM USABILITY**

| Statement | Level of agreement | | | | |
| --- | --- | --- | --- | --- | --- |
|  | Strongly agree | Agree | Neutral | Disagree | Strongly disagree |
| 27. I think that I would like to use the EMR system frequently |  |  |  |  |  |
| 28. I found the system unnecessarily complex |  |  |  |  |  |
| 29. I think the system is easy to use |  |  |  |  |  |
| 30. I think that I would need the support of a technical person to be able to use this system. |  |  |  |  |  |
| 31. I found the various functions in this system were well integrated |  |  |  |  |  |
| 32. I think there is too much inconsistency in this system. |  |  |  |  |  |
| 33. I would imagine that most people would learn to use EMR system very quickly. |  |  |  |  |  |
| 34. I found the system very cumbersome to use |  |  |  |  |  |
| 35. I felt very confident using the EMR system |  |  |  |  |  |
| 36. I needed to learn a lot of things before I could get going with this system |  |  |  |  |  |

**SECTION G: INFORMATION QUALITY**

| Statement | Level of agreement | | | | |
| --- | --- | --- | --- | --- | --- |
|  | Strongly agree | Agree | Neutral | Disagree | Strongly disagree |
| 37. The report is in a useful format |  |  |  |  |  |
| 38. The information is clear |  |  |  |  |  |
| 39. The information is complete |  |  |  |  |  |
| 40. The information is precise |  |  |  |  |  |
| 41. The information is timely |  |  |  |  |  |
| 42. The information is secured |  |  |  |  |  |
| 43. The information is up to date |  |  |  |  |  |

**SECTION H: SERVICE QUALITY**

| Statement | Level of agreement | | | | |
| --- | --- | --- | --- | --- | --- |
|  | Strongly agree | Agree | Neutral | Disagree | Strongly disagree |
| 44. IT staff understand our need |  |  |  |  |  |
| 45. The EHR training is enough |  |  |  |  |  |
| 46. Computer access is enough |  |  |  |  |  |
| 47. I’m satisfied with user guideline |  |  |  |  |  |
| 48. There is easy access to technicians’ support |  |  |  |  |  |
| 49. There is no frequently power interruption |  |  |  |  |  |
| 50. There is back up power in the unit |  |  |  |  |  |
| 51. The system is rarely subjected to system problems and crashes |  |  |  |  |  |
| 52. Software problem get fixed in acceptable time frame |  |  |  |  |  |

**SECTION I: eHEALTH LITERACY**

| Statement | Level of agreement | | | | |
| --- | --- | --- | --- | --- | --- |
|  | Strongly agree | Agree | Neutral | Disagree | Strongly disagree |
| 53. I know where to find helpful health resources on the Internet |  |  |  |  |  |
| 54. I know how to find helpful health resources on the Internet |  |  |  |  |  |
| 55. I know how to use the Internet to answer my questions about health |  |  |  |  |  |
| 56. I know how to use the health information I find on the Internet to help me |  |  |  |  |  |
| 57. I have the skills I need to evaluate the health resources I find on the Internet |  |  |  |  |  |
| 58. I can distinguish high-quality health resources from low-quality health resources on the Internet |  |  |  |  |  |
| 59. I feel confident in using information from the Internet to make health decisions |  |  |  |  |  |

**THANK YOU**
